# From femtoseconds to minutes: time-resolved macromolecular crystallography at XFELs and synchrotrons

**DOI:** 10.1107/S2059798323011002

**Published:** 2024-01-24

**Authors:** Nicolas Caramello, Antoine Royant

**Affiliations:** a Structural Biology Group, European Synchrotron Radiation Facility, 1 Avenue des Martyrs, CS 40220, 38043 Grenoble CEDEX 9, France; b Hamburg Centre for Ultrafast Imaging, Universität Hamburg, HARBOR, Luruper Chaussee 149, 22761 Hamburg, Germany; c Université Grenoble Alpes, CNRS, CEA, Institut de Biologie Structurale (IBS), 71 Avenue des Martyrs, CS 10090, 38044 Grenoble CEDEX 9, France; ESRF, France

**Keywords:** time-resolved serial crystallography, synchrotrons, XFELs, structural photobiology, reaction-intermediate states, bacteriorhodopsin, cryo-trapping

## Abstract

Over the last decade, the development of time-resolved serial crystallography (TR-SX) at X-ray free-electron lasers (XFELs) and synchrotrons has allowed researchers to study phenomena occurring in proteins on the femtosecond-to-minute timescale, taking advantage of many technical and methodological breakthroughs. Protein crystals of various sizes are presented to the X-ray beam in either a static or a moving medium. Photoactive proteins were naturally the initial systems to be studied in TR-SX experiments using pump–probe schemes, where the pump is a pulse of visible light. Other reaction initiations through small-molecule diffusion are gaining momentum. Here, selected examples of XFEL and synchrotron time-resolved crystallography studies will be used to highlight the specificities of the various instruments and methods with respect to time resolution, and are compared with cryo-trapping studies.

## Abstract 2

This review constitutes an overview of the current status of time-resolved crystallography performed at synchrotrons and XFELs on timescales ranging from femtoseconds to minutes. Methods, potential biases, instruments and examples are presented and compared with those for the cryo-trapping of reaction-intermediate states.

## 1. Introduction

Most X-ray macromolecular crystallography (MX) experiments aim at determining the static structures of macromolecules to understand their architecture or the fine details of interactions within an active site or of bound ligands. However, the knowledge of a static structure is not sufficient to understand the mechanism enabling the function of the corresponding macromolecule. For this purpose, structure determination of reaction-intermediate states is highly desirable. While a static structure describes a molecule in equilibrium with its environment, reaction-intermediate states are metastable species, *i.e.* out of equilibrium, with a finite lifetime. The duration of data collection is thus of utter importance for structure determination of the latter. Consequently, the observation of reaction intermediates has long been out of the reach of crystallography. In the 1980s, this started to change with the parallel development of Laue diffraction and cryo-crystallography. The goal of developing Laue diffraction was to decrease the exposure time of single frames from minutes to seconds and beyond (Moffat, 2019 ▸). The increase in the brightness of synchrotron sources progressively enabled the use of shorter trains of pulses, until single 100 ps bunches could be used. This led to intricate studies on the mechanism of the photolysis and rebinding of carbon monoxide to the haem of sperm whale myoglobin (Schotte *et al.*, 2003 ▸) and on the photocycle of the cytosolic blue-light photoreceptor PYP (photoactive yellow protein) from the bacterium *Halorhodo­spira halophila* (Jung *et al.*, 2013 ▸). Unfortunately, the polychromatic nature of the technique placed strenuous requirements on crystal quality and the probed photoreaction had to be fully reversible on a rather short timescale. In parallel, cryo-crystallography was developed, originally as a way to stabilize very fragile crystals (Hope *et al.*, 1989 ▸). The use of cryo-crystallography surged in the early 1990s as a way to extend the crystal lifetime in the X-ray beam (Garman & Schneider, 1997 ▸). Flash-cooling a crystal at cryogenic temperatures had the added benefit of freezing protein dynamics, which paved the way for the development of protocols to trap reaction-intermediate states, which were collectively coined ‘cryo-trapping’ techniques (Bourgeois & Royant, 2005 ▸). All techniques attempting to determine the structure of reaction-intermediate states, either at room temperature by time-resolved Laue diffraction or at cryogenic temperature by classical monochromatic crystallography, were termed ‘kinetic crystallography’ at the time.

The field of kinetic crystallography was rejuvenated in the early 2010s with the advent of X-ray free-electron lasers (XFELs), which were predicted to impact MX (Neutze *et al.*, 2000 ▸). The demonstration of the ability to determine the structure of a macromolecule from microcrystals using a femtosecond X-ray pulse opened the possibility of time-resolved MX (TR-MX) with unprecedented time resolution and a wider range of biological targets (Chapman *et al.*, 2011 ▸). XFELs fostered a wave of technical (sample-delivery methods in particular) and methodological (data-set reconstruction from single frames in particular) developments that gave birth to the so-called ‘serial femtosecond crystallography’ (SFX) technique (Schlichting, 2015 ▸). It was quickly realized that these advances could be used on synchrotron beamlines, bringing about serial synchrotron (or millisecond) crystallo­graphy (SSX/SMX; Nogly *et al.*, 2015 ▸). The major success of serial crystallography (SX) has been achieved in time-resolved crystallography, in the form of TR-SFX at XFELs (Orville, 2020 ▸) and TR-SSX at synchrotrons (Pearson & Mehrabi, 2020 ▸).

This article reviews the current status of TR-SFX and TR-SSX with a comparison to cryo-trapping methods. An overview of instruments, pitfalls, methods and guidelines is presented, with a focus on selected examples studying protein dynamics on timescales ranging from femtoseconds to minutes.

## 2. The various methods to catch reaction intermediates

### 2.1. How to start a reaction

Kinetic crystallography has essentially focused so far on light-triggerable reactions, as the speed of light permits a quasi-simultaneous activation of all molecules within a crystal, provided that the crystal is not too thick and the light fluence is high enough. Synchronization of reactions occurring in every molecule of the crystal is crucial to minimize the problem of intermediate-state mixtures at a given time point. Unfortunately, only a small fraction of proteins are light-activatable (less than 0.5%; Monteiro *et al.*, 2021 ▸). Reactions that rely on substrate and cofactor recruitment by a protein active site generally have to be initiated by the diffusion of small molecules within the solvent channels of a crystal. As a consequence, crystal size and diffusion rates are key parameters affecting synchronization which can greatly hinder kinetic crystallography experiments (Schmidt, 2013 ▸). A hybrid approach has been proposed to alleviate the diffusion issue by using a photocaged substrate or cofactor (which are inert until a light pulse releases the chemical cage) to enable productive recruitment of the substrate or cofactor into the active site (Monteiro *et al.*, 2021 ▸).

A number of stratagems have been developed in X-ray crystallography to facilitate reaction initiation. One method exploits the fact that X-rays induce photoelectrons within the crystal bulk solvent to initiate redox reactions at cryogenic temperatures, in a controlled manner using the principle of composite data sets, with the support of UV–Vis absorption microspectrophotometry (Berglund *et al.*, 2002 ▸). With more sensitive detectors, full data sets can be used to provide dose points (Rose *et al.*, 2022 ▸). Other exciting possibilities include the use of electric field stimulation to target specific protein motions (Hekstra *et al.*, 2016 ▸) and that of a nanosecond infrared (IR) laser to induce temperature jumps (Wolff *et al.*, 2023 ▸). Wolff and coworkers used this approach in TR-SFX experiments to probe the inhibition mechanism of an enzyme, lysozyme, by comparing the dynamics of the apo and inhibitor-bound forms 20 ns and 200 µs after a temperature jump.

### 2.2. Strategies to catch the structure of intermediate states

**Figure 1:**
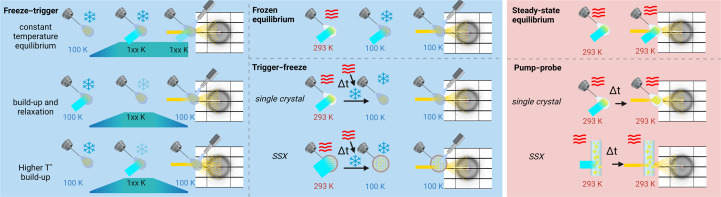
Overview of common strategies that can be used to capture the structure of reaction intermediates using light as the reaction trigger. Protocols implicating cryogenic temperature data collection are represented with a blue background: to block a reaction initiated at room temperature once an equilibrium is reached (‘frozen equilibrium’), or before it is reached (‘trigger–freeze’) or to limit the progress of a reaction by initiating it at cryogenic temperature (‘freeze–trigger’). Protocols implicating room-temperature data collection are represented on a red background: to maintain a reaction at equilibrium (‘steady-state equilibrium’) or to catch intermediates as the reaction proceeds (‘pump–probe’). For simplicity, only protocols relying on photoactivation are depicted here. Meaning of symbols: cyan rectangles, UV–visible actinic light; yellow rays, X-rays; red waves, room temperature; blue snowflake, cryogenic temperature

The various data-collection strategies that can be used to determine the structure of an unstable reaction intermediate can be grouped into two categories. In the first category, the ‘cryo-trapping’ group, a crystal is flash-cooled to cryogenic temperature in order to alter the reaction kinetics by either quenching or slowing down protein dynamics before or after initiation of the reaction. Diffraction data collection is ultimately performed at low cryogenic temperature. In the second category, the ‘room temperature’ group, data collection is performed at room temperature according to a time scheme that is consistent with the expected reaction kinetics. Representative examples of these strategies using light as a reaction trigger are displayed in Fig. 1 ▸.

Cryo-trapping strategies can be divided into three sub­groups for light-triggered reactions. In the first group, the reaction is initiated by illumination and quenched at some point under continuous illumination (‘frozen equilibrium’); see, for instance, Fedorov *et al.* (2003 ▸) and Gotthard *et al.* (2019 ▸). In the second group, the reaction is triggered at room temperature and then quenched by flash-cooling at various time delays (‘trigger–freeze’ protocol). This approach can be used on single crystals (Basu & Murakami, 2013 ▸) or on a slurry of microcrystals (Suga *et al.*, 2019 ▸), eventually requiring an SX technique for data collection such as *MeshAndCollect* (Zander *et al.*, 2015 ▸). The third group involves starting from flash-cooled crystals, to which a temperature profile is applied (‘freeze–trigger’ protocol), for example to populate a photo-equilibrium at a given constant cryogenic temperature under constant illumination (*T* = 110 K in Edman *et al.*, 1999 ▸; *T* = 85 K in Kort *et al.*, 2004 ▸) or to initiate the reaction at low temperature and populate an early intermediate, and then give energy to the system so that the reaction can progress to a later intermediate. Additionally, one may want to raise the temperature and initiate the reaction within an active site with more thermal energy, and collect data at this temperature (*T*_illumination/data collection_ = 150 K in Sorigué *et al.*, 2021 ▸) or back at the original temperature (*T*_illumination_ = 150 K in Edman *et al.*, 2004 ▸).

Room-temperature strategies can essentially be divided into two groups. In the first, less used group, the reaction is initiated by illumination until a steady-state equilibrium is reached and data collection is then performed whilst still under illumination (‘steady-state equilibrium’; see, for instance, Crosson & Moffat, 2002 ▸). In the second group, the experiment is performed under a ‘pump–probe’ sequence. The pump (*i.e.* laser) pulse is applied at a given time and the probe (*i.e.* X-ray) pulse is initiated at various delays afterwards. This strategy can be applied to single crystals or to many different microcrystals, to a point where only one still diffraction frame is acquired at most from a microcrystal. Many examples of pump–probe experiments are described in Sections 3, 5, 6 and 7.

### 2.3. How to monitor the progress of a reaction within the crystal

Kinetic crystallographic experiments can generate a multitude of diffraction data. For cryo-trapping experiments, significant variations of the experimental strategy (temperature profile, illumination scheme) need to be probed. For TR-SX experiments, many orders of time magnitude have to be explored in order to isolate intermediates of interest. Performing these experiments without prior knowledge could prove to be very time-consuming both for data acquisition and analysis. For experiments on coloured proteins, UV–Vis absorption spectroscopy appears to be a suitable complementary technique to rapidly provide information on successful cryo-trapping strategies or time points of particular interest by identifying intermediates with specific spectroscopic signatures (von Stetten *et al.*, 2015 ▸; Makita *et al.*, 2023 ▸). These methods can potentially be extended to noncoloured samples by the use of Raman (Bui *et al.*, 2014 ▸) or IR (Nomura *et al.*, 2021 ▸; Mous *et al.*, 2022 ▸) vibrational spectroscopy.

Because the kinetics of a reaction can differ greatly for a protein between the solution and the crystalline state (see Section 4.1), it is of prime importance to perform these spectroscopic experiments directly on the crystals that will be used during the diffraction experiment, and possibly also in solution. A number of instruments and facilities have been built for this purpose, in general close to synchrotron sources, such as the *ic*OS (*in crystallo* Optical Spectroscopy) Laboratory at the ESRF (von Stetten *et al.*, 2015 ▸), the SpectroLab at the SLS (Pompidor *et al.*, 2013 ▸) and beamline BL26B1 at SPring-8 (Rose *et al.*, 2022 ▸), which took over the online microspectrophotometry capability of BL26B2 (Sakaguchi *et al.*, 2016 ▸).

**Figure 2:**
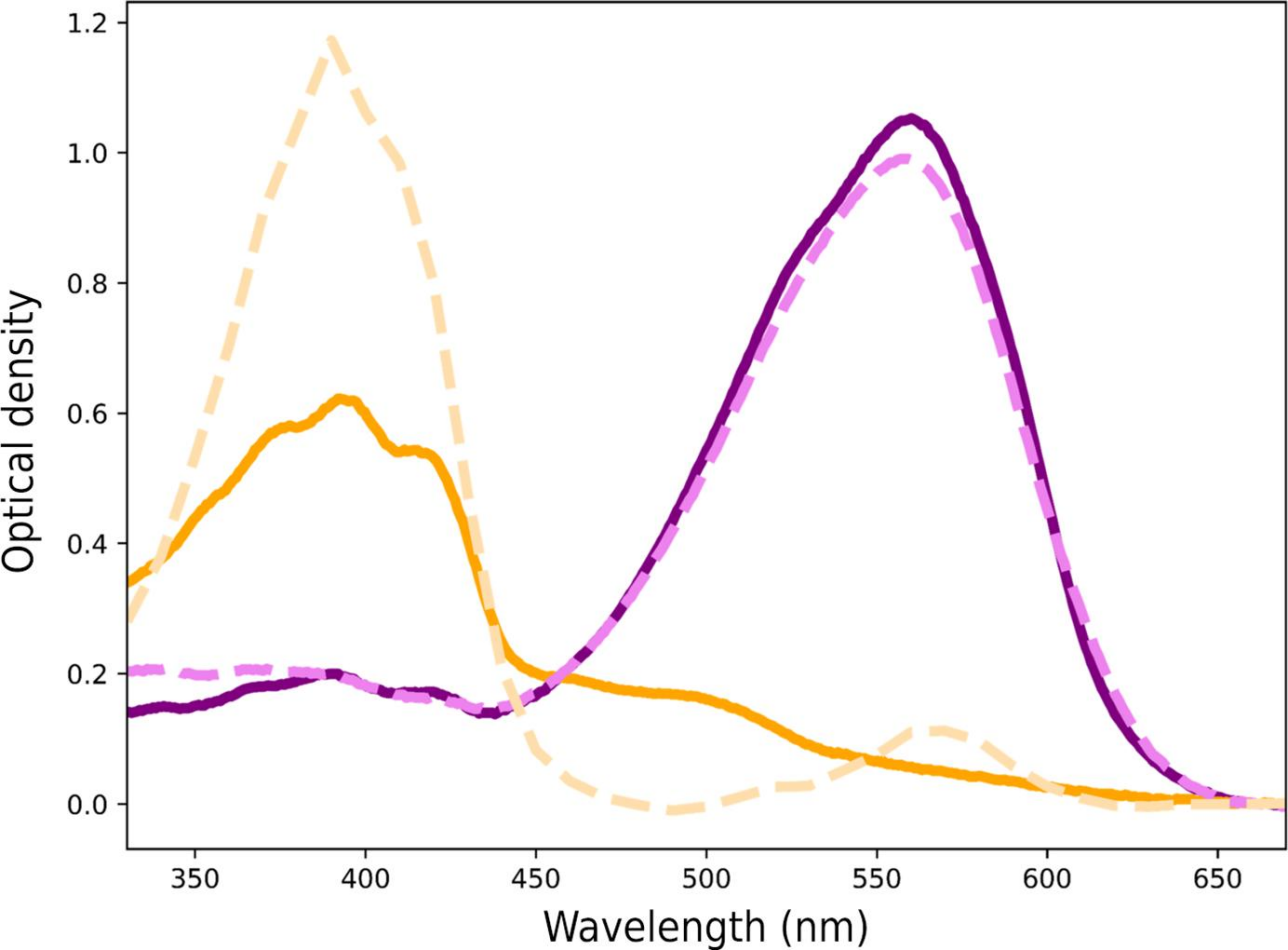
*In crystallo* UV–Vis absorption spectra (solid lines) compared with spectra in solution (dashed lines) for the ground state (purple, fuschia) and the cryo-trapped M state (orange, light orange) in the photocycle of *Bc*XeR (data reprised from Kovalev *et al.*, 2023 ▸). The difference in respective peak heights between the solution and crystal M-state spectra may be due to a polarization effect of the crystal.

In particular, the use of UV–Vis absorption spectroscopy is helpful in cryo-trapping experiments to validate the presence of the expected reaction intermediate in sufficient proportion. Suboptimal protocols may lead to the mixing of several intermediates, potentially with the expected intermediate as a minor population. Kovalev and coworkers used the *ic*OS Laboratory to devise a cryo-trapping protocol of the M state in the photocycle of xenorhodopsin from *Bacillus coahuilensis* (*Bc*XeR; Kovalev *et al.*, 2023 ▸). The M state was obtained using the frozen equilibrium strategy (illumination for 1 s with a 532 nm laser followed by flash-cooling). The UV–Vis absorption spectrum of the corresponding crystal shows that it is predominantly populated in the M state (Fig. 2 ▸), thereby validating the cryo-trapping protocol for data collection on the beamline.

## 3. Existing facilities and instruments for TR-SFX and TR-SSX

TR-SFX experiments have been performed at most existing XFELs. The first experiments were performed at the Linac Coherent Light Source (LCLS), Stanford, California, USA, which opened in 2009. There are essentially two beamlines at the LCLS hosting TR-SFX experiments: CXI (Coherent X-ray Imaging), on which the first experiment was performed with microsecond time resolution on co-crystals of Photosystem I and ferredoxin (Aquila *et al.*, 2012 ▸), and MFX (Macromolecular Femtosecond Crystallography), whose first experiment was performed with hundreds of microseconds time resolution on crystals of Photosystem II (Kern *et al.*, 2018 ▸). The other pioneering XFEL facility is SACLA in Harima Science Garden City, Hyogo Prefecture, Japan, which opened in 2011, with the first TR-SFX study being performed on beamline BL3 on crystals of bacteriorhodopsin (Nango *et al.*, 2016 ▸). Beamline BL2 was used for the first time on channel­rhodopsin crystals (Oda *et al.*, 2021 ▸). Three other facilities opened later: PAL-XFEL, Pohang, South Korea in 2016, SwissFEL, Villigen, Switzerland in 2017 and EuXFEL, Schenefeld, Germany, also in 2017. The first TR-SFX experiment at SwissFEL took place on beamline ARAMIS-ALVRA using crystals of the light-driven sodium pump KR2 on a wide timescale from hundreds of femtoseconds to tens of milliseconds (Skopintsev *et al.*, 2020 ▸). The first TR-SFX experiment at EuXFEL took place on the SPB/SFX instrument using crystals of PYP at time points in two time ranges: 10–80 ps and 0.89–2.67 µs (Pandey *et al.*, 2020 ▸). Finally, the new XFEL facilities LCLS-II (at Stanford) and SHINE-XFEL (in Shanghai) will soon be available, as well as beamline ARAMIS-CRISTALLINA at SwissFEL.

Cryo-trapping experiments can be performed on essentially any MX synchrotron beamline, provided that the sample temperature can be precisely controlled. Polychromatic (or Laue) TR-SSX has been performed on the BioCARS beamline at the Advanced Photon Source (APS), Argonne, Illinois, USA to visualize β-lactam cleavage by a metallo-β-lactamase using ten snapshots ranging from 20 to 4000 ms (Wilamowski *et al.*, 2022 ▸). Monochromatic TR-SSX experiments have so far been reported on a handful of beamlines. PX1 at the Swiss Light Source (SLS), Villigen, Switzerland was used with a grease injector to track the structure of late intermediates in the photocycle of bacteriorhodopsin (Weinert *et al.*, 2019 ▸). The TR-SSX capability of I24 at Diamond Light Source (DLS), Didcot, United Kingdom was demonstrated with a study of the photoswitching of the fluorescent protein rsEospa observed 1 ms after light triggering (Baxter *et al.*, 2022 ▸). P14.2 (T-REXX) at PETRA III, Hamburg, Germany was the first beamline to be specifically designed for TR-SSX. Its first application was the design of the hit-and-return (HARE) data collection scheme to determine three reaction snapshots for fluoro­acetate dehalogenase between 30 ms and 2 s (Schulz *et al.*, 2018 ▸). The PETRA III P14 MX beamline has also been used for TR-SSX, with submillisecond time resolution (Kovalev *et al.*, 2023 ▸). Similarly, steady-state SSX experiments have been performed on the XALOC beamline at ALBA, Barcelona, Spain, also on the sodium pump KR2 (Kovalev *et al.*, 2020 ▸). The build-up of a steady-state equilibrium could be monitored with a time resolution of 63 ms using a serial oscillation crystallography approach on beamline ID30A-3 (MASSIF-3) at the ESRF, Grenoble, France (Aumonier *et al.*, 2020 ▸). Finally, dedicated beamlines have recently been designed and constructed at fourth-generation synchrotron sources for the purpose of TR-SSX: ID29 at the ESRF and MicroMAX at MAX IV, Lund, Sweden.

## 4. Potential biases to be considered

### 4.1. Crystal packing

The first obvious artefact in TR-MX arises from the very fact that the technique requires the molecule to be crystallized, and thus it resides in a significantly different environment than in solution or in the native cellular environment, as molecules are in close interaction with symmetry-related neighbours through crystal contacts and experience a different level of hydration. As a consequence, the crowded but ordered environment in a crystal differs from the dilute environment often used in solution studies and even from the crowded, yet most often unordered, environment of a cell. Protein dynamics components may be either hindered or exacerbated, calling for a verification of whether a reaction proceeds in a similar or in a significantly different manner. For instance, it has been shown by both electronic and vibrational transient absorption spectroscopies that the photocycle of PYP differs *in crystallo* from that in solution (Konold *et al.*, 2020 ▸). The nature of a few intermediate states differs (the presence of an additional state at an early stage and the absence of an intermediate state at a late stage of the photocycle *in crystallo*), and the rise and decay time constants of equivalent intermediates may vary by more than one order of magnitude. The differences are proposed to be explained by a combination of reduced hydration, different viscosity and confinement in the crystal lattice. In another example, the nature of the intermediate in the photocycle of an LOV (light–oxygen–voltage-sensing) domain is conserved between the solution and the crystalline state, but the kinetics of its decay are significantly affected (Aumonier *et al.*, 2022 ▸). The relaxation decay time constant increases from 6 s in solution to 40 s *in crystallo*. This difference is likely to stem from crystal contacts, but may also originate from reduced hydration that could slow down thioether-bond rupture.

### 4.2. Temperature

During the golden age of cryo-crystallography (2000–2010), flash-cooling of crystals at cryogenic temperature was thought to minimally affect the structure of proteins. However, flash-cooling usually requires the addition of cryoprotectant small molecules to the crystal mother liquor, which may specifically bind to the active site of a protein (Bukhdruker *et al.*, 2023 ▸) and thus affect its function, which can be detected by *in crystallo* optical spectroscopy control experiments in favourable cases (von Stetten *et al.*, 2012 ▸). Moreover, James Fraser and collaborators determined room-temperature crystallo­graphic structures of proteins and compared them with the cryogenic structures. They found evidence that even though the secondary structure was conserved, a significant proportion of side-chain conformers were altered (Fraser *et al.*, 2009 ▸, 2011 ▸). This means that the conformational landscape of a protein active site can potentially suffer from artefacts stemming from flash-cooling, and the validity of cryo-trapped intermediate states should always be questioned as a matter of principle.

Alternatively, freeze–trigger approaches rely on the assumption that the intermediate states populated at low temperatures resemble those of the physiological reaction. This assumption should always be validated by complementary methods, as the conformational landscape may drive the low-temperature photoreaction off the physiological pathway.

### 4.3. Radiation damage

**Figure 3:**
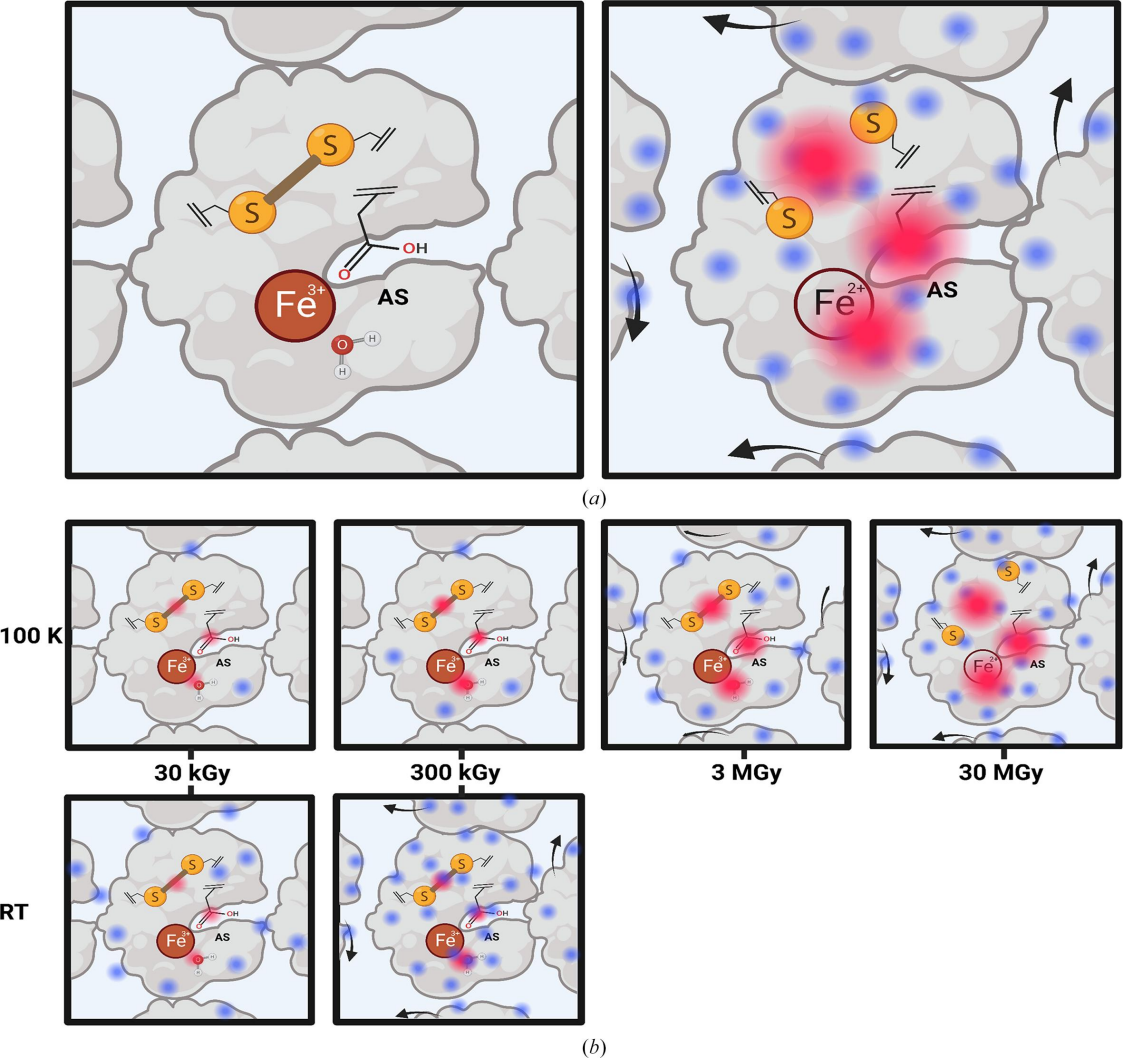
Global versus specific radiation damage. Top row, left: a single crystal of a hypothetical protein is depicted with three specific radiation damage-sensitive groups: a disulfide bridge, an oxidized metal cation and a residue with a carboxylate group located in the active site (AS). Top row, right: the crystal is affected by global damage (blue dots) with various effects, in particular that of perturbating crystal contacts upon X-ray-induced protein movement within the cell (black arrows), leading to a loss of diffraction resolution, an increase in mosaicity and increased *B* factors. Specific damage (red dots) develops on the aforementioned radiosensitive chemical groups, leading to disulfide-bridge reduction (rupture), metal-cation reduction and residue decarboxylation. Middle row: illustration of the damage dose scale at cryogenic temperature. Global damage builds up slowly, while specific damage builds up relatively rapidly, so as to be visible at low doses in Fourier difference maps calculated between successive data sets 1 and *n*, *F*_obs(*n*)_ − *F*_obs(1)_, and at high doses in *F*_calc_ − *F*_obs_ and 2*F*_calc_ − *F*_obs_ maps. The maximum acceptable dose is typically that of the Garman limit (30 MGy). Bottom row: illustration of the damage dose scale at room temperature: both types of damage build up on a similar dose scale, complicating the precise identification of specific damage. The maximum acceptable dose for a single crystal is a few hundreds of kilograys, *i.e.* typically one hundredth of the Garman limit.

Radiation damage is an issue inherent to X-ray crystallo­graphy (Garman & Weik, 2023 ▸). Global damage and specific damage can be distinguished. Global damage affects the diffraction properties of a given crystal (decrease in resolution and increase in mosaicity, unit-cell volume and scaling *B* factor between successive data sets), which can all be visualized on diffraction spots (location on the image, shape, intensity; *i.e.* in reciprocal space). On the other hand, specific damage affects specific chemical groups (carboxylate groups, disulfide bonds, metallic cations), which can all be visualized in electron-density maps; *i.e*. in real space) (Fig. 3 ▸). Specific damage was identified by cryogenic data collection at third-generation synchrotrons around the year 2000 (Burmeister, 2000 ▸; Weik *et al.*, 2000 ▸; Ravelli & McSweeney, 2000 ▸). It was soon realized that the structural changes stemming from specific radiation damage could be mixed up with, or mistaken for, those from intermediate states trapped at cryogenic temperature, and thus hamper precise identification of the latter. This was first observed for bacteriorhodopsin (Matsui *et al.*, 2002 ▸) and later for other systems, some of which contained metallic cations in their active site: horseradish peroxidase (Berglund *et al.*, 2002 ▸), superoxide reductase in complex with ferricyanide (Adam *et al.*, 2004 ▸) and a bacterial photosynthetic reaction centre (Baxter *et al.*, 2004 ▸). In the cases of metal-containing enzymes, the controlled X-ray-induced reduction of the cations is elegantly used to reveal the subtle details of the response of the protein to the change in oxidation state of its metal cofactor that are involved in catalysis. It thus became of utter importance in kinetic crystallography to perform control experiments in order to differentiate structural changes that can be attributed to the build-up of a reaction-intermediate state from those that would develop as a result of specific radiation damage, either of the resting-state structure or on the intermediate-state structure itself.

The revival of room-temperature crystallography at the end of the 2000s (Fraser *et al.*, 2009 ▸, 2011 ▸) prompted the evaluation of how global radiation damage could affect room-temperature structures (Southworth-Davies *et al.*, 2007 ▸; Leal *et al.*, 2013 ▸) and eventually concluded that full data sets could be recorded in a few hundreds of kilograys from a single crystal. Analysis of these data sets concluded that unlike with cryogenic data sets, specific radiation damage could not be observed clearly (Russi *et al.*, 2017 ▸; Gotthard *et al.*, 2019 ▸). It was further suggested that only a small fraction of the molecules in the crystals that contributed to diffraction were affected by this phenomenon (Gotthard *et al.*, 2019 ▸). This hypothesis was rationalized by showing that specific and global damage occur at similar dose scales at room temperature, but at cryogenic temperature global damage develops much more slowly than its counterpart, constituting a decoupling of these two types of radiation damage. While specific damage is difficult to grasp in single-crystal data sets, the fine dose slicing permitted by SSX led to the clear visualization of specific damage to microcrystals (Schubert *et al.*, 2016 ▸; Mora *et al.*, 2020 ▸). One significant bottom line of these observations is that for oscillation MX at a synchrotron, the level of scrutiny required of an intermediate-state structure determined at cryogenic temperature may be relaxed for single-crystal data collection at room temperature, for which specific damage will not be apparent.

The rapid loss of crystalline order in Photosystem I nanocrystals placed in an intense 100 fs X-ray pulse suggested that specific radiation damage would be an afterthought at XFELs (Barty *et al.*, 2012 ▸). Yet, it has been shown that long pulses (80 fs) may induce what amounts to specific damage to iron–sulfur clusters in ferredoxin by direct photo-ionization of these heavy atoms when compared with 30 fs pulses (Nass *et al.*, 2015 ▸). Moreover, pump–probe SFX experiments using two pulses separated by a delay varying between 18 and 112 fs showed the build-up of specific damage to disulfide bonds and the protein backbone in lysozyme and thaumatin (Nass *et al.*, 2020 ▸). This has been turned into a trick to study the structural effects of iron(III) photoreduction in the iron-binding protein FutA using a delay time of 33 ms between an attenuated and an unattenuated XFEL pulse (Bolton *et al.*, 2023 ▸). The effects of microsecond pulses in TR-SSX have only started to be studied, and should soon provide insights into the extent of both global and specific damage in this uncharted time regime.

### 4.4. Light fluence

The first TR-SFX studies that were performed did not specifically address the possibility that the fluence of the pump laser illuminating the sample could affect the physiological photoreaction that was probed. As crystals of light-sensitive proteins are highly concentrated in chromophores, the penetration depth of the pump-laser photons is limited to a few micrometres to a few tens of micrometres, depending on the extinction coefficient of the chromophore at the particular wavelength used. Thus, it is tempting to increase the laser power until a meaningful signal can be visualized in a difference electron-density map calculated between a dark and a light data set. The first TR-SFX study to address the potential problem of excessive light fluence used ultrafast visible and infrared spectroscopy to probe the effect of increasing power densities on the photocycle of bacteriorhodopsin (Nass Kovacs *et al.*, 2019 ▸). It was concluded that above a certain threshold the chromophore can sequentially absorb two photons, leading to the excitation of a nearby tryptophan residue. However, no change in the presented structural data obtained with a moderate fluence was ascribed to the artefact identified spectroscopically at higher fluences. Miller and coworkers then proposed that in order to be sure that only a single-photon process is probed in a TR-SFX experiment, the exciting laser power should be kept at a level ensuring an excitation fluence of less than a photon per chromophore within the 1/*e* absorption depth (Miller *et al.*, 2020 ▸; Besaw & Miller, 2023 ▸). The bottom line is that it is of critical importance to evaluate as precisely as possible how many photons are absorbed by crystals in a TR-SFX experiment (Grünbein *et al.*, 2020 ▸). Also, it is highly advisable to perform power-titration experiments, ideally via both diffraction and spectroscopy (Barends, Stauch *et al.*, 2022 ▸). New instruments have been built to address the same concern for future TR-SSX experiments (Engilberge *et al.*, 2024 ▸). One should note that a significant fraction of the incident light is scattered by the sample-carrying medium, which should be estimated and considered in the calculations. Ideally, these power-titration experiments should reveal a linear photoactivation regime, in which additional photons increase the yield of the photoreaction but do not steer it onto artefactual pathways. Performing TR-SX experiments at the maximum laser fluence in the identified linear regime will contribute to optimizing the number of necessary indexed images, thus making the most of the allocated beamtime.

## 5. Bacteriorhodopsin: an emblematic example of fast TR-MX

**Figure 4:**
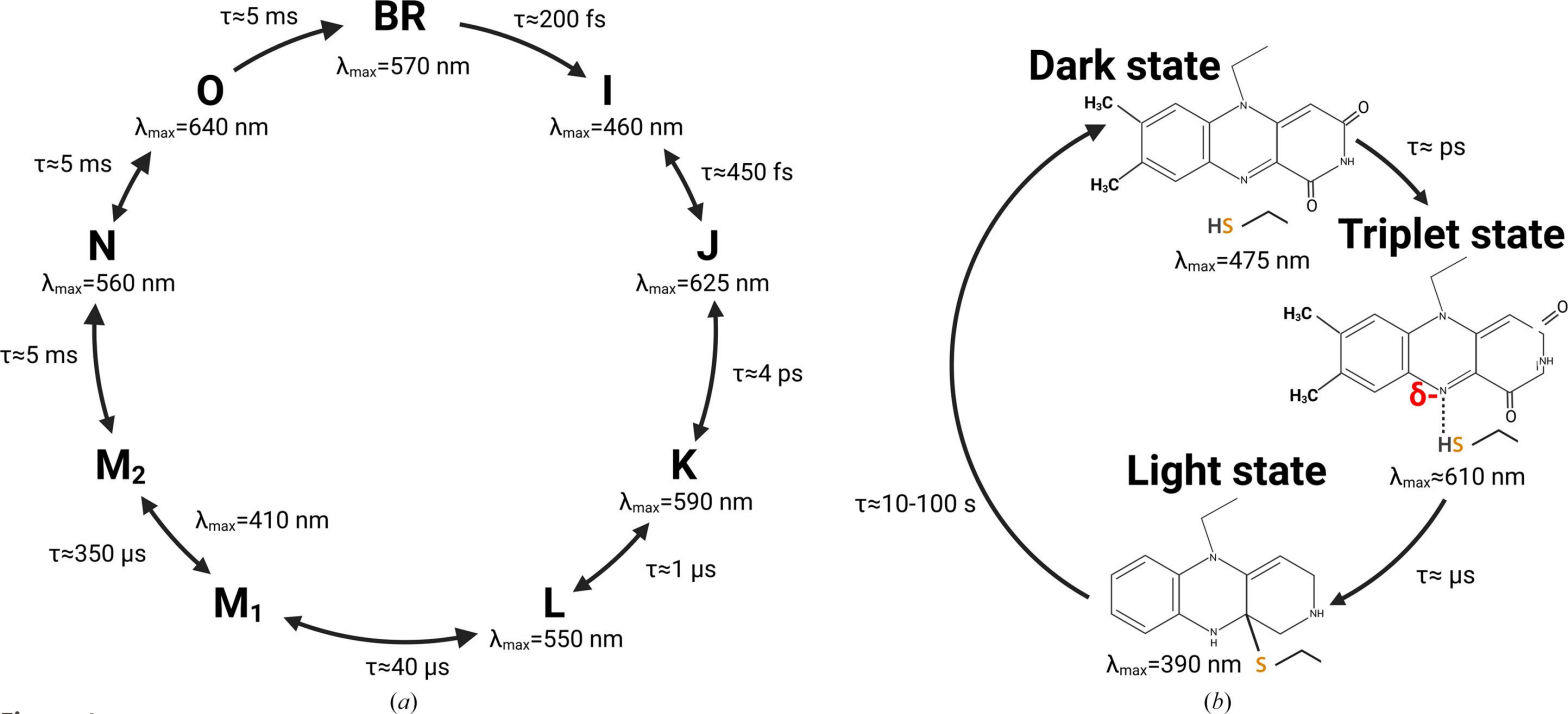
Photocycles of (*a*) bacteriorhodopsin and (*b*) an LOV domain.

Bacteriorhodopsin (BR) is a light-driven proton pump that is found in the membrane of the halophilic archaeon *Halobacterium salinarum* (Oesterhelt & Stoeckenius, 1971 ▸), which has served as a paradigm for both the spectroscopic and structural characterization of membrane proteins (Ottolenghi & Sheves, 1995 ▸; Haupts *et al.*, 1999 ▸). BR is composed of seven transmembrane helices, and its chromophore retinal is covalently bound through a Schiff-base linkage to a lysine residue (Lys216) located in the middle of the seventh helix (helix G). Upon the absorption of a photon by its chromophore retinal, the protein adopts a succession of spectroscopic states characterized by specific UV–Vis absorption maxima before returning to the ground state, forming a photocycle (Fig. 4 ▸ *a*). The successful crystallization of bacteriorhodopsin in lipidic cubic phases (Landau & Rosenbusch, 1996 ▸) led to the determination of its crystallographic structure at increasing resolutions (Pebay-Peyroula *et al.*, 1997 ▸; Luecke *et al.*, 1998 ▸; Belrhali *et al.*, 1999 ▸), paving the way for high-resolution structures of intermediate states in its photocycle. Because of the significant mosaicity of the crystals, the room-temperature approach using Laue diffraction never materialized and the first period of structural characterization of the BR photocycle relied entirely on cryo-trapping (Wickstrand *et al.*, 2015 ▸).

### 5.1. Cryo-trapping studies

The first crystallographic study of a BR photoreaction intermediate was that of the K state populated at low temperature (110 K) upon illumination with green light (Edman *et al.*, 1999 ▸). The structural changes are confined to the environment close to the chromophore, with signs of retinal isomerization, disordering of a water molecule that was previously in a hydrogen-bond interaction with the Schiff-base nitrogen, and movement of neighbouring residues. A later study on the L state (Royant *et al.*, 2000 ▸), which was populated at a higher cryogenic temperature (170 K), revealed that the perturbation of the hydrogen-bond network had progressed towards the extracellular side of the protein. At the same time as this study, a structure of the M state was obtained (Sass *et al.*, 2000 ▸). These three studies performed on the wild-type protein provided an initial structural picture of the events following light absorption by the chromophore retinal and leading to its deprotonation (Kühlbrandt, 2000 ▸). A number of studies on BR mutants and also on wild-type BR with different trapping protocols followed, yielding conflicting results, thus providing a blurred picture of what could really be precisely achieved by cryo-trapping methods (Wickstrand *et al.*, 2015 ▸). Another complicating factor was the realization that specific radiation damage affected the structure of the ground and K states of BR at low dose (Matsui *et al.*, 2002 ▸; Borshchevskiy *et al.*, 2011 ▸, 2014 ▸). A recent effort towards maximizing diffraction resolution provided an improved picture of the K, L and M states, most particularly regarding the presence of hydrogen bonds (Borshchevskiy *et al.*, 2022 ▸). Nonetheless, the controversies regarding the structure of cryo-trapped intermediates in the BR photocycle set the stage for time-resolved studies at room temperature.

### 5.2. XFEL studies

After a feasibility study on the M state (Nogly *et al.*, 2016 ▸), the first breakthrough XFEL study used a nanosecond pump laser and aimed to probe the structural events ranging from the K state, which builds up in picoseconds and is thus well present at nanoseconds, to the onset of the large structural changes occurring to helices that are expected in the M2 state after a build-up over several hundreds of microseconds (Nango *et al.*, 2016 ▸). At 16 ns, isomerization of the retinal is completed, displacing a tryptophan residue on helix F and initializing the perturbation of the hydrogen-bond network bridging the chromophore and the extracellular side. The perturbation progressively develops to prepare the irreversible proton transfer from the retinal Schiff-base nitrogen to the primary acceptor (Asp85) through the transient ordering of a water molecule and the concomitant release of a proton into the extracellular medium. Conversely, structural changes in the cytoplasmic part develop in the microsecond to millisecond regime, setting up the conditions for retinal reprotonation from the cytoplasm. However, the latest time point at 1.7 ms did not reveal large changes of the cytoplasmic part, calling into question whether the crystal form would not completely hinder them.

**Figure 5:**
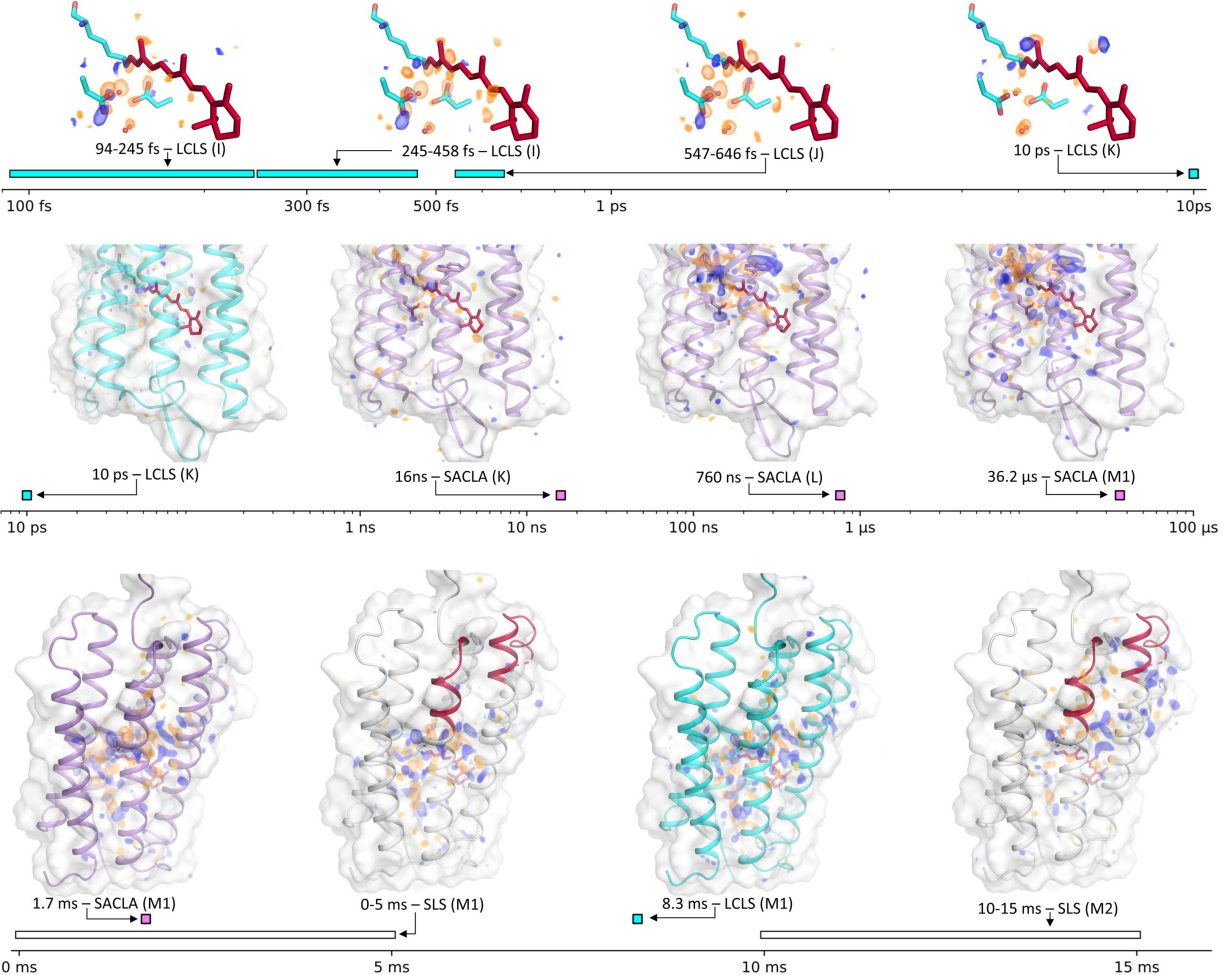
Overview of structural changes in the photocycle of BR visualized by TR-SFX and TR-SSX over 13 orders of magnitude. Fourier difference maps (yellow, negative; blue, positive) are contoured at the 3.9σ level in the top row and at the 3.0σ level in the middle and bottom rows, and are superimposed on the corresponding ground-state structure. Data are reprised from a TR-SFX study performed at SACLA (lilac; Nango *et al.*, 2016 ▸), a TR-SFX study performed at the LCLS (cyan; Nogly *et al.*, 2018 ▸) and a TR-SSX study performed at the SLS (white; Weinert *et al.*, 2019 ▸). The parts of helices E, F and G depicted in red in the SLS structures are those that undergo large-scale displacement or disordering in the late phase of the photocycle.

The second XFEL effort aimed to understand the very first steps in the photocycle of bacteriorhodopsin using a femtosecond laser instead (Nogly *et al.*, 2018 ▸). This led to the visualization of the response of the protein and chromophore to the absorption of a green photon from hundreds of femtoseconds to 10 ps, covering the build-up and decay of the first three intermediates I, J and K, with the addition of an 8.33 ms time point acting as a reference for the M state, which is very consistent with the 1.7 ms time point recorded in the previous experiment (Fig. 5 ▸). The various snapshots are consistent with a mechanism in which the electronically excited chromophore initially samples possible isomerization geometries (as suggested by the number of negative peaks along the retinal chain at *t* < 458 ps), before C13=C14 is selected (J state; 457–636 fs, rotated bond), until a twisted isomerized chromophore builds up in the K state at 10 ps. The collective motions of the primary acceptor Asp85, the neighbouring Asp212 and close water molecules during this process are proposed to illustrate how these chemical groups favour the stereoselectivity and efficiency of retinal isomerization. A parallel study proposed a very similar view of the first steps of the response of retinal to light absorption in bacteriorhodopsin (Nass Kovacs *et al.*, 2019 ▸). A useful lesson from this study is the spectroscopic evidence that multiphoton absorption may occur in TR-SFX experiments, calling for a better control of light fluence (see Section 4.4).

### 5.3. Synchrotron studies

BR has often been used as a target in the development of SX experiments at synchrotrons (SSX experiments; Nogly *et al.*, 2015 ▸; Zander *et al.*, 2015 ▸). Appropriately, it became one of the first systems to successfully be used in TR-SSX experiments. Building on the TR-SFX results, Weinert and coworkers designed an experimental setup on beamline PX1 of the SLS that was able to track structural changes in the BR photocycle by illuminating a moving grease jet of crystals for 5 ms with a CW class 3R green laser diode (520 nm; Weinert *et al.*, 2019 ▸) every 200 ms. The EIGER detector was operated at 200 Hz, so that the first frame of a cycle corresponded to crystals under illumination (the 0–5 ms frame), while the 39 later frames corresponded to increasing delays in a pump–probe scheme from 5–10 ms to 195–200 ms. While the diffraction data were at moderate resolution, the 5–10 ms time point fittingly showed structural features associated with the M state visualized in previous studies (Fig. 5 ▸). However, the following time point, 10–15 ms, revealed the build-up of an open form of the protein, with large-scale movements or disordering of the cytoplasmic parts of helices E–G, with the largest displacement (∼9 Å) occurring at the tip of helix F. These changes must be associated with the N state only, as the O state has been shown to hardly be populated in crystals of BR (Efremov *et al.*, 2004 ▸).

### 5.4. TR-SFX and TR-SSX of other microbial and nonmicrobial rhodopsins

The intensive structural characterization of the BR photocycle has paved the way for the investigation of other microbial rhodopsins, which can exhibit many functions other than outward proton pumping (Rozenberg *et al.*, 2021 ▸). The light-driven sodium pump KR2 from *Krokinobacter eikastus* was investigated at SwissFEL with time delays between 800 fs and 20 ms (Skopintsev *et al.*, 2020 ▸). The light-driven chloride pump *Nm*HR from *Nonlabens marinus* has been studied by two different groups (Yun *et al.*, 2021 ▸; Mous *et al.*, 2022 ▸). In the latter study, a combination of TR-SFX and TR-SSX (between 10 ps and 300 µs at SwissFEL and between 2.5 and 55 ms at SLS) was used to structurally describe the whole photocycle of *Nm*HR, particularly the dynamics of the transported chloride ion. Finally, the photocycle of the light-driven bacterial inward proton pump xenorhodopsin from *Bacillus coahuilensis* (*Bc*XeR) was studied at PETRA III by TR-SSX at submillisecond resolution, with the L state and M state probed with time delays of 250–750 µs and 7.5–15.0 ms, respectively (Kovalev *et al.*, 2023 ▸).

Finally, visual rhodopsins, which are not homologous to microbial rhodopsins, have started to be studied by TR-SFX. The structure of the first intermediate in the photocycle of bovine rhodopsin (from *Bos taurus*), bathorhodopsin, was obtained at SwissFEL using a delay of 1 ps after excitation by a femtosecond laser, which showed that the retinal is in a distorted conformation that has cancelled a significant number of the interactions with the protein present in the dark state (Gruhl *et al.*, 2023 ▸).

## 6. LOV domains: an emblematic example of slow TR-MX

Light–oxygen–voltage-sensing (LOV) domains are a subclass of Per–ARNT–Sim (PAS) sensor domains, which are present in all kingdoms of life (Taylor & Zhulin, 1999 ▸). In photosynthetic organisms, they constitute the light-sensing module of the blue-light photoreceptor phototropin, which controls various processes implicated in photosynthesis condition optimization, such as phototropism (Christie, 2007 ▸). An LOV domain uses a flavin mononucleotide (FMN) as a chromophore. Upon the absorption of a blue-light photon, the excited state FMN* first decays into a triplet state within nanoseconds, and then forms a covalent adduct with a nearby conserved cysteine within a few microseconds (Swartz *et al.*, 2001 ▸), which we call the ‘light’ state (Fig. 4 ▸ *b*). In the second LOV domain of phototropin, LOV2, the formation of this covalent bond induces a series of structural rearrangements that culminate in the unfolding of a helix located after the C-terminal part of the LOV domain, J_α_, eventually activating a serine/threonine protein kinase domain, which serves as the effector domain of the photoreceptor (Harper *et al.*, 2003 ▸). The light state of a LOV domain returns to the dark state within tens of seconds. The distinct lifetimes of these intermediate states call for time-resolved studies involving ultrafast to slow crystallographic techniques.

### 6.1. Cryo-trapping and room-temperature steady-state studies

The first attempt to determine the structure of the light state of a plant LOV2 domain was performed using the photo­stationary (steady-state) method at room temperature, *i.e.* by constantly illuminating the crystal before and during the whole X-ray data collection (Crosson & Moffat, 2002 ▸). Apart from the thioether bond between the FMN and the protein, a number of side-chain rearrangements could be observed next to the chromophore due to the change in hydrogen bonding, particularly for a conserved glutamine residue next to the N5 atom of the FMN.

A different approach was used to determine the light state of a green alga LOV1 domain (Fedorov *et al.*, 2003 ▸). Here, a trigger–freeze approach was used: the crystals were illuminated in crystal trays before flash-cooling in liquid nitrogen. The resulting structure exhibited specific radiation damage to the thioether bond, thus requiring the merging of data from two different crystals.

Further crystallographic studies focused on the transmission of the light signal through structural change of the adjacent helix J_α_. This was attempted for both a bacterial (Möglich & Moffat, 2007 ▸) and a plant (Halavaty & Moffat, 2007 ▸) LOV domain; in both cases the trigger–freeze approach was used. For the latter study, photostationary conditions were also used during room-temperature data collection. In both studies, movement of the J_α_ helix, or part of it, was observed in the light-state structures, but no genuine unfolding was visualized.

### 6.2. Monitoring of light-state build-up by a time-resolved multi-crystal oscillation approach

**Figure 6:**
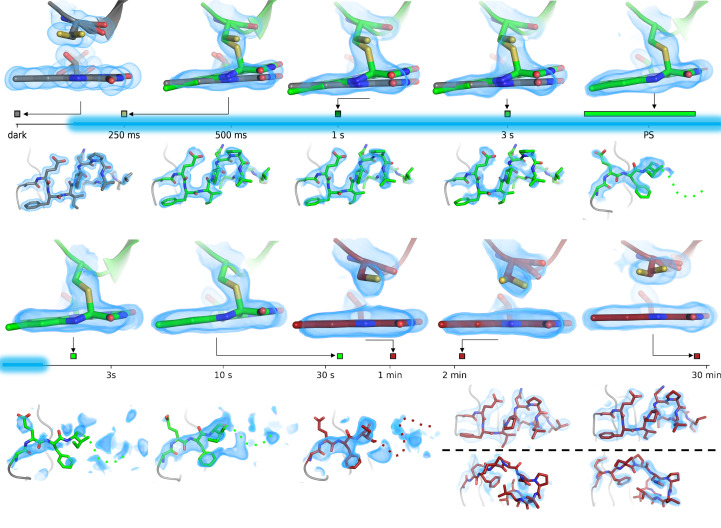
Time-resolved structural changes in an LOV2 domain over four orders of time magnitude (from 63 ms to 27 min) as observed in Aumonier *et al.* (2020 ▸) and Aumonier *et al.* (2022 ▸). The sequence starts with the dark state (grey), which then proceeds to a photostationary equilibrium (PS, green) with the light state under continuous illumination (light blue in timeline). After illumination has been stopped, the light state relaxes back to the dark state, yet in a different crystallographic state (red). The upper part of each panel features the cysteine residue and the FMN chromophore that engage in a thioether bond, and the lower part represents the C-terminal part. All 2*F*_calc_ − *F*_obs_ maps are contoured at the 1.0σ level, except that of the dark-state structure (grey, top left), for which the contour level is 1.5σ.

The light state of LOV domains builds up in microseconds, rendering its mechanistic study by TR-SSX a challenge. In order to detail the progressive build-up of structural features accounting for the dark-state to light-state transition, an experimental protocol was devised by taking advantage of oscillation data sets recorded on single crystals of a plant LOV2 domain recorded under continuous illumination, the initiation of which is synchronized with the start of data collection (Aumonier *et al.*, 2020 ▸). In order to slow down light-state build-up in the crystal at the population level, the photon budget was limited by tuning down the power of the exciting blue light-emitting diode (LED). Complementary UV–Vis absorption spectroscopy performed off-line allowed identification of the LED power that gave rise to a build-up with a time constant of about 1 s. Approximately 100 data collections were performed on single crystals with the same data-collection strategy: 1000 images of 0.5° rotation and 4.2 ms exposure each, resulting in a total collection time of 4.2 s. Each data set was separated into 15-image sub-data sets, which were merged together using a clustering algorithm, resulting in full data sets corresponding to 66 time points: 0–63 ms, 64–126 ms, …, 4095–4158 ms. The significant number of adjacent time points made it easier to identify and model structural changes in the light state, namely on five residues surrounding the FMN chromophore, including the cysteine implicated in thioether-bond formation (see the four first time points in the first timeline in Fig. 6 ▸ *a*: *t* < 0 s, *t* ≃ 250 ms, *t* ≃ 1 s and *t* ≃ 3 s). The time constants derived from the various refined occupancies of protein stretches around these five residues and of the FMN averaged to 1.45 ± 0.14 s, which is qualitatively close to the value of 0.89 s separately derived by time-resolved UV–Vis absorption spectroscopy. This experiment demonstrated that the TR-SOX technique (time-resolved serial oscillation crystallography) could be used on a finite number of crystals to structurally probe a time-dependent phenomenon occurring at room temperature, which here was the increase in the light-state population during the build-up of a steady-state equilibrium under continuous illumination.

### 6.3. Monitoring light-state relaxation using a time-resolved single-crystal oscillation approach

The end point of the experiment described in the previous section is the establishment of a steady-state equilibrium in a crystal of LOV2. The relatively slow decay time of the light state (Fig. 4 ▸ *b*) prompted Aumonier and coworkers to monitor how it relaxed by taking advantage of the fast acquisition rate of a Dectris EIGER X 4M detector and recording full oscillation data sets in only 1.2 s [400 images of 0.3° rotation (120° total rotation) and 3 ms acquisition time] on different crystals at various time points after termination of the illumination (Aumonier *et al.*, 2022 ▸). The structure of the first time point (*t* < 0, *i.e.* under constant illumination; the last structure in the first timeline in Fig. 6 ▸) is that of LOV2 in a steady-state equilibrium composed predominantly of the light state and characterized by disorder of the C-terminus of the protein. As soon as the illumination is stopped, the occupancy of the thioether bond starts to decrease and electron density starts to build up on the C-terminus (the first three structures in the second timeline in Fig. 6 ▸). Shortly after 60 s, the diffraction data cannot be unambiguously reduced in a tetragonal space group but only in an orthorhombic space group, revealing the formation of a non-crystallographic dimer, which differs in the conformation of its C-terminus. The C-terminus of one monomer (upper row) folds into the helical conformation adopted in the dark-state structure at the beginning of the experiment, *i.e.* without any illumination, while that of the other monomer (lower row) eventually folds into a hook-shaped conformation (the final two structures in the second timeline). The whole relaxation is achieved 27 min after the end of illumination. This work constitutes a TR-MX study of a phenomenon occurring on a timescale of minutes to tens of minutes, which was probed with a time resolution of 1.2 s, demonstrating the feasibility of slow time-resolved diffraction studies on single crystals.

## 7. Other light-activatable proteins

### 7.1. Photoactive yellow protein (PYP)

PYP is a cytosolic blue-light photoreceptor from the phototrophic bacterium *Halorhodospira halophila* and is implicated in negative phototaxis. It uses a 4-hydroxycinnamic acid molecule as a chromophore. Because PYP became the hallmark of time-resolved Laue crystallography, it was naturally chosen as one of the first targets to be investigated by TR-SFX. The remaining gaps in the structural understanding of its photoreaction were the events surrounding the *cis*/*trans* isomerization step, which was expected to take place in the hundreds of femtoseconds regime, much shorter than the 100 ps time resolution of Laue crystallography. The first TR-SFX study aimed to compare the quality of the produced difference electron-density maps with those obtained by Laue crystallography (Tenboer *et al.*, 2014 ▸). To this end, the well defined snapshots at 1 µs (corresponding to the most visible changes ascribed to the pR_1_ and pR_2_ states of the photocycle; that is, the movement of the S atom of the thioether bond linking the chromophore to the protein) and 10 ns (corresponding to a more challenging situation in which the three intermediate states I_CT_, pR_1_ and pR_2_ coexist and their deconvolution thus requires particularly good-quality data) were later chosen (Pande *et al.*, 2016 ▸). In a second step, the 142–1023 fs time domain was sampled in order to visualize the series of events leading to, and following, *cis*/*trans* isomerization, which is proposed to occur between 500 and 650 fs. An additional time point at 3 ps served to characterize the fully relaxed intermediate. Overall, these studies have completed the structural description of the full PYP photocycle *in crystallo*, which starts with light-induced chromophore isomerization and proceeds to hydrogen-bond network re­organization. This eventually leads to the unfolding of two N-terminal helices, which constitutes the signalling state of the photoreceptor.

### 7.2. Photoenzymes

For a long time, only two types of light-driven enzymes (photoenzymes) had been recognized as such: photolyases, which are DNA-repair enzymes that convert pyrimidine dimers into a pair of pyrimidine bases under exposure to UV light (Sancar, 2003 ▸), and light-dependent protochorophyllide oxido­reductase (LPOR), which catalyses one of the last steps in chlorophyll biosynthesis (Heyes & Hunter, 2005 ▸). These two canonical photoenzymes were later joined by fatty-acid photodecarboxylase (FAP), which uses blue light to convert fatty acids to hydrocarbons (Sorigué *et al.*, 2017 ▸). Since the crystallographic structure of LPOR has been solved in the presence of the NADPH cofactor but without the substrate (Zhang *et al.*, 2019 ▸), time-resolved studies of its mechanism will have to wait until suitable crystals can be grown. The enzymatic photoenzyme of FAP has been studied by a wealth of biophysical techniques, including both cryo-trapping and time-resolved crystallographic approaches (Sorigué *et al.*, 2021 ▸). The TR-SFX experiment probed events occurring 20 ps to 2 µs after light excitation. For photolyases, the mechanism of photoreduction of the FAD cofactor, which amounts to enzyme activation before the catalysis of damaged DNA repair can happen, was first deciphered by TR-SFX for a CPD (cyclobutane pyrimidine dimer) photolyase (Maestre-Reyna *et al.*, 2022 ▸) and by TR-SSX for a (6–4) photolyase (Cellini *et al.*, 2022 ▸). In a second step, the whole repair mechanism of a CPD lesion by a photolyase was revealed from picoseconds to hundreds of microseconds (Maestre-Reyna *et al.*, 2023 ▸; Christou *et al.*, 2023 ▸). It consists of the transfer of one electron from the FAD cofactor to the damaged DNA, the sequential breaking of two covalent bonds, the rearrangement of the various chemical groups involved in the reaction and, finally, the back-flipping of the two repaired DNA bases, which leads to dissociation of the enzyme–DNA complex.

A loosened definition of a photoenzyme may also apply to other systems (Björn, 2018 ▸). For instance, Photosystem II, the large membrane-protein complex that is responsible for the splitting of water during photosynthesis, has been extensively studied by (TR-)SFX, most notably because of the extreme sensitivity of its manganese cluster to X-ray-induced reduction precluding reliable (TR-)MX studies at synchrotrons. After an initial low-resolution TR-SFX study suggesting an elongation of the cluster (Kupitz *et al.*, 2014 ▸), a markedly higher resolution study uncovered structural changes occurring 10 ms after the sequential absorption of two photons, suggesting the formation of an oxo-bridge with the cluster (Suga *et al.*, 2017 ▸). The chemical nature of this bridge could later be precisely assessed as an oxyl/oxo species thanks to the higher resolution enabled by a cryo-trapping approach (Suga *et al.*, 2019 ▸). The full redox cycle of PSII (Kok’s clock) was first investigated by a TR-SFX study using multiple excitation schemes and varied time delays (Kern *et al.*, 2018 ▸), and then by a more recent study which focuses on the last steps of the redox cycle (Bhowmick *et al.*, 2023 ▸).

### 7.3. Phytochromes

Phytochromes are red and far-red light photoreceptors whose kinase activity is implicated in key cellular processes such as growth, germination, heat and light sensing, and phototropism in plants and fungi (Cheng *et al.*, 2021 ▸), photoprotection in nonphotosynthetic bacteria (Davis *et al.*, 1999 ▸) and the synthesis of the photosynthetic apparatus in photosynthetic bacteria (Giraud & Verméglio, 2008 ▸). The structure of the chromophore-binding domain of bacteriophytochrome from *Deinococcus radiodurans* (*Dr*Bph) revealed that the biliverdin chromophore is covalently bound to the PAS domain and inserted within the GAF domain (Wagner *et al.*, 2005 ▸). This first structure of truncated phytochrome paved the way for structural studies to understand the conversion between the red-absorbing Pr and far-red-absorbing Pfr states of the photoreceptor. Using a longer construct of *Dr*Bph that includes the additional phytochrome-specific domain PHY, Takala and coworkers showed that the GAF and PHY domains were separated by a structural element formed of two short β-strands belonging to the PHY domain: the PHY-tongue. Using the ‘frozen equilibrium’ cryo-trapping strategy, they revealed that the light-induced Pr-to-Pfr conversion consists of the refolding of the tongue into an α-helix (Takala *et al.*, 2014 ▸). The same team attempted to visualize the details of the transition by TR-SFX by recording time points 1 and 10 ps after light excitation (Claesson *et al.*, 2020 ▸). This study demonstrated that the twist of the D ring upon light-induced isomerization of the chromophore drives a sequence of events (dissociation of the pyrrole water molecule and movement of the A ring and of an aspartate residue) that ultimately leads to ultrafast backbone movement and thus to destabilization of the PHY-tongue. They also performed a TR-SFX study on a different bacteriophytochrome from the myxobacterium *Stigmatella aurantiaca* at the later time points of 5 ns and 33 ms (Carrillo *et al.*, 2021 ▸). They observed a more pronounced isomerization of the chromophore and displacement of the PHY domain both through the PHY-tongue and the long α-helix connecting the GAF and PHY domains.

### 7.4. Phototransformable fluorescent proteins

The function of a fluorescent protein (FP) is to absorb photons around a certain energy (centred around the maximum peak of its absorption spectrum) using its chromophore, which is promoted to an excited state and then returns to the ground state by re-emitting secondary photons of lower energy. The efficiency of an FP is quantified by the ratio of emitted photons to absorbed photons, which is called the fluorescence quantum yield QY (0 ≤ QY ≤ 1). De-excitation from the excited state occurs via radiative and non­radiative pathways, and the latter are minimized in an efficient FP. As a consequence, the chromophore of an efficient FP is constrained by a rigid environment, and thus the mechanism of fluorescence does not rely on atomic movements that could easily be observed by TR-SFX during the fluorescence lifetime, which is of the order of several nanoseconds. However, there is a class of FPs whose complex spectroscopic properties rely on the transformation of the chemical nature of the chromophore via isomerization or covalent-bond breakage: the phototransformable FPs (PTFPs). The study of their phototransformation mechanism is possible by TR-SFX.

The first example of a PTFP to be studied was the reversibly photoswitchable protein rsEGFP2 (Coquelle *et al.*, 2018 ▸). Crystals of rsEGFP2 in the (non-fluorescent) off-state were excited with a 400 nm femtosecond laser, and data collection was performed 1 and 3 ps later. At 1 ps after laser excitation the chromophore exhibits a mixture of two conformations: one planar close to that of the off-state (model P) and one twisted halfway between the configurations of the off- and on-states (model T). At 3 ps model P has relaxed and there is a mixture of model T and a conformation resembling the on-state. A second study probed a later time point at 10 ns to validate that chromophore isomerization has occurred on this timescale (Woodhouse *et al.*, 2020 ▸). Another group chose the same protein but with a chemically modified chromophore, in which a Cl atom has been added to the terminal ring, in order to use TR-SFX to investigate whether the chromophore-isomerization process occurs via the hula-twist or the one-bond-flip pathway (Fadini *et al.*, 2023 ▸). Structural changes were probed 300 fs, 600 fs, 900 fs, 5 ps, 100 ps and 1 µs after laser excitation and first revealed that the Cl atom remains on the same side of the ring, demonstrating that isomerization occurs through the hula-twist mechanism. Surprisingly, traces of the isomerized chromophore are already present at 300 fs. Also, a constrained intermediate builds up at 600 fs and then decays by 5 ps. Isomerization is then completed by 100 ps. A TR-SSX study was applied to another reversibly photoswitchable FP (rsFP), rsEospa, to probe the nature of the isomer produced during 1 ms of exposure to laser illumination for different isomers at pH values ensuring different protonation states (Baxter *et al.*, 2022 ▸). Finally, the same group as in the two latter examples evolved the rsFP EosFP into rsKiiro with improved photochemical properties (which include a high photochemical quantum yield of photoisomerization) and diffraction resolution at room temperature (better than 1.5 Å for microcrystals at XFELs) in order to amplify the signal contained in the Fourier difference maps. They used a two-pulse pump–dump excitation scheme (at 400 and 515 nm, respectively) to investigate whether the ultrafast (subpico­second) protein dynamics form part of the photoisomerization process. By using a single-pulse excitation scheme as a control, they were able to disprove this (Hutchison *et al.*, 2023 ▸).

## 8. Examples of TR-MX studies of biological systems relying on ligand or substrate delivery

TR-SX experiments initially focused on photoreactions, for which reaction triggering is initiated with a visible laser pulse, thus minimally complicating the sample environment. In order to broaden the range of targets, sophisticated substrate/cofactor-delivery systems had to be developed and fitted within a crowded experimental setup. There are essentially two ways of initiating a substrate/cofactor-dependent reaction.

The first and more versatile method relies on the diffusion of a small molecule within the channels of a protein crystal, either by mixing of solutions (for a flow of crystals) or soaking (for a stationary crystal, to which a cofactor/substrate solution is delivered). It is worth noting that the diffusion of substrates and cofactors into microcrystals occurs on the high-microsecond, low-millisecond timescale at best (Makinen & Fink, 1977 ▸; Schmidt, 2013 ▸; Pandey *et al.*, 2021 ▸), which prevents access to fast to ultrafast events. Various types of sample-delivery setups have been developed. Firstly, a microfluidic-based mixing capacity can be positioned upstream of a viscous or liquid sample injector (Wang *et al.*, 2014 ▸; Calvey *et al.*, 2016 ▸; Doppler *et al.*, 2022 ▸). Samples can be probed within the microfluidic device itself, for instance the 3D-printed microfluidic chip 3D-MiXD (Monteiro *et al.*, 2020 ▸). Alternatively, crystals can be deposited onto a moving tape (TapeDrive system) after rapid liquid mixing has occurred (Beyerlein *et al.*, 2017 ▸; Zielinski *et al.*, 2022 ▸). The BITS (comBination of Inject-and-Transfer System) sample-delivery method combines the advantages of the sample-injection and fixed-target approaches by injecting a pre-mixture of crystals and solutions through a needle tip onto an ultraviolet ozone-treated polyimide film held on a translation stage, horizontal and vertical motions of which permit scanning of the whole film by the X-ray beam (Lee *et al.*, 2022 ▸). While these techniques rely on the mixing of solutions, an alternative consists of delivering droplets of substrate/cofactor solution directly onto a crystal sitting on a sample holder, which amounts to crystal soaking. In the first such example, crystals are loaded onto a fixed-target chip onto which a substrate/cofactor solution is sprayed: this technique has been named LAMA (Liquid Application Method for time-resolved Analyses; Mehrabi, Schulz, Agthe *et al.*, 2019 ▸). Of note, this approach requires the installation of a humidity-control chamber around the sample environment. Similarly, the drop-on-drop method consists of delivering bursts of picolitre-sized substrate/cofactor drops onto crystals positioned on a moving tape (Butryn *et al.*, 2021 ▸). All of these techniques have primarily been developed for room-temperature time-resolved applications, but they could be adapted for cryo-trapping approaches for timescales above the typical flash-cooling time (∼1–100 ms depending on the crystal size) using the MMQX (millisecond mix-and-quench crystallography; Clinger *et al.*, 2021 ▸) and spitrobot (Mehrabi *et al.*, 2023 ▸) approaches.

The second method, as already mentioned in Section 2.1, relies on the photo-induced cleavage of a protective group in a so-called ‘photocaged’ substrate/cofactor (Monteiro *et al.*, 2021 ▸). If the photocaged molecule has been co-crystallized or soaked with the crystals prior to the experiment, the limiting factor here is not the small-molecule diffusion time through the solvent channels but the ligand-release time after the decaging light pulse.

**Figure 7:**
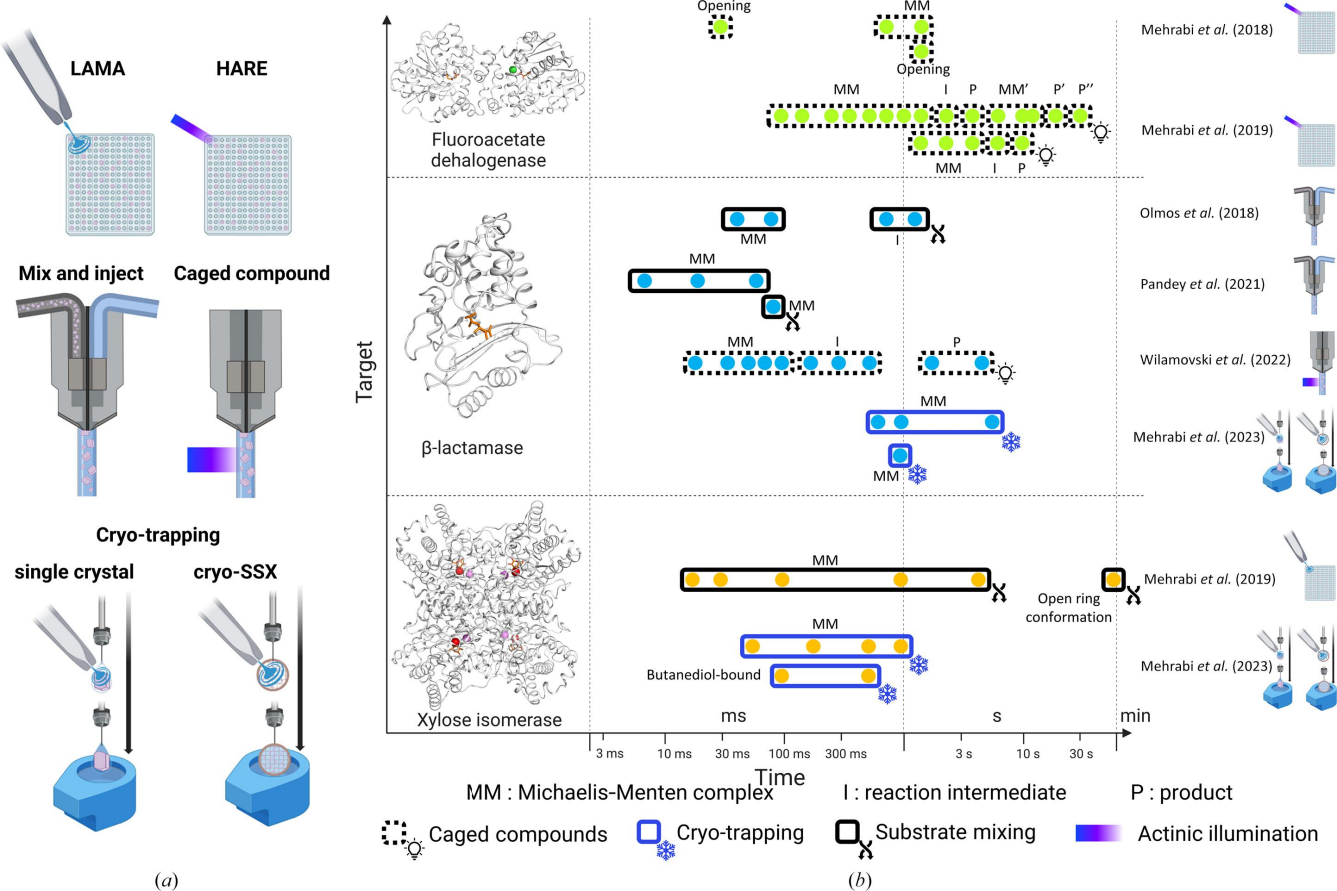
Diffusion-based time-resolved experiments. (*a*) Strategies enabling the initiation of a reaction by the diffusion of a substrate or a cofactor. (*b*) Overview of studies conducted on emblematic targets: fluoroacetate dehalogenase (depicted using PDB entry 5k3a; Mehrabi, Schulz, Dsouza *et al.*, 2019 ▸), β-lactamase (depicted using PDB entry 7bh5; Butryn *et al.*, 2021 ▸) and xylose isomerase (depicted using PDB entry 8aw8; Mehrabi *et al.*, 2023 ▸). Colour code: protein secondary structure, white; substrate, orange; ions, green (chloride ion), red [magnesium(II) ion] and lilac [manganese(II) ion].

Three main examples of diffusion-based reactions studied by TR-SX have focused attention over the past years. The enzyme fluoroacetate dehalogenase (FAcD) was first used to develop the ‘hit-and-return’ (HARE) method, which enables TR-SSX for time resolutions ranging from milliseconds to tens of seconds (Fig. 7 ▸ *a*; Schulz *et al.*, 2018 ▸). Using caged fluoro­acetate, the binding of its natural substrate by FAcD was studied using an experimental protocol that minimizes the data-acquisition time. At 30 ms, active-site opening is observed for one of the two monomers in the asymmetric unit. Between 752 and 2052 ms, the substrate-bound state, or Michaelis–Menten (MM) complex, is progressively populated. At 2052 ms, the active site of the second monomer starts to open as well (Fig. 7 ▸ *b*). Using the same approach, the same authors greatly extended the time range to ∼30 s to observe four catalytic cycles of enzyme turnover (three for monomer *A* and one for monomer *B*; Mehrabi, Schulz, Dsouza *et al.*, 2019 ▸). For monomer *A*, the MM complex is observed between 188 and 2052 ms. At 2256 ms, the formation of a covalent intermediate (C) is observed. The product (P) is observed at 4512 ms and is released by 6156 ms. Two further cycles are subsequently observed. A different kinetic is observed for monomer *B*, suggesting that substrate access to the active site is allosterically controlled.

The LAMA technique (Fig. 7 ▸ *a*) was developed by the same authors using xylose isomerase (XI; Mehrabi, Schulz, Agthe *et al.*, 2019 ▸). A solution of glucose was sprayed onto XI crystals sitting in the wells of a fixed-target chip and diffraction data sets were recorded at approximate time points of 15 ms, 30 ms, 100 ms, 1 s, 4.5 s and 60 s. Binding of the substrate is maximal at 100 ms. XI appears to be idle at 1 and 4.5 s, but the glucose ring is observed to be open at 60 s, demonstrating enzymatic activity (Fig. 7 ▸ *b*). XI was also used to demonstrate the cryo-trapping capability of the spitrobot (Mehrabi *et al.*, 2023 ▸). They first showed that the cryoprotectant 2,3-butanediol binds preferentially to glucose within 50 and 500 ms (*i.e.* before flash-cooling). In the absence of 2,3-butanediol, they were able to observe full occupancy of glucose binding at 50, 250, 500 and 1000 ms.

Next, various antimicrobial resistance proteins of the β-lactamase (β-lac) type were studied using many of the abovementioned techniques and methods. Olmos and coworkers used an mix-and-inject approach to visualize the binding of an antibiotic to β-lac (time points of 30 and 100 ms) and its subsequent cleavage (500 ms and 2 s) (Olmos *et al.*, 2018 ▸). A follow-up study observed binding at shorter time points for the same antibiotic (5, 10 and 50 ms) and that of an inhibitor at 66 ms (Pandey *et al.*, 2021 ▸). A caged Zn^2+^ approach was used at BioCARS with Laue serial diffraction to probe ten time points of the reaction of a metallo-β-lactamase with the antibiotic moxalactam from 20 to 4000 ms (Wilamowski *et al.*, 2022 ▸). For the five time points between 20 and 100 ms, substrate binding is observed (Fig. 7 ▸ *b*). From 150 to 500 ms, a hydrolysed moxolactam intermediate is observed. The product then relaxes within the active site by 2 s and remains in place until 4 s at least. Finally, an activity-impaired β-lac was also used to test the spitrobot, which allowed the authors to characterize the inhibitor-bound structure by cryo-SSX from microcrystals (flash-cooled after 1 s) and the covalent binding of ampicillin in single crystals at time points of 0.5, 1 and 5 s (Mehrabi *et al.*, 2023 ▸).

## 9. Concluding remarks

### 9.1. Interest of cryo-trapping studies

Compared with cryo-trapping, time-resolved crystallo­graphy appears to be the better-suited approach to probe a reaction since the conditions are closer to the physiological conditions. However, these experiments, which are most often performed in a serial manner, are challenging in terms of sample quantity, experimental knowhow (particularly the control of laser fluence) and diffraction data processing (Barends, Bhattacharyya *et al.*, 2022 ▸). Also, diffraction resolution is often limited at room temperature. Cryo-trapping methods potentially imply a number of drawbacks: flash-cooling and cryoprotection artefacts (for trigger–freeze strategies), altered conformational landscape (for freeze–trigger strategies), specific radiation damage (for synchrotron experiments) and limited time resolution, which is dictated by the flash-cooling time, which is just below 1 ms in the best case for microcrystals (Clinger *et al.*, 2021 ▸). However, they do benefit from three significant advantages: (i) production of diffraction data usually at much higher resolution than at room temperature, (ii) ease of diffraction data analysis (for the single-crystal case) and (iii) potentially permitting the accumulation of higher intermediate-state occupancy. The latter can help to overcome the main limitation in kinetic crystallography, which is the difficulty of identifying the structural features of a poorly populated intermediate state, especially when this difficulty is aggravated by non-isomorphism between crystals. While keeping in mind the limitations of cryo-trapping, high-resolution intermediate-state structures can be used to interpret lower resolution data obtained at room temperature. One final recommendation to navigate between time-resolved approaches and the many cryo-trapping approaches is to perform characterization experiments at various temperatures and with different light-excitation schemes (light-source type, wavelength peak or range, duration), possibly via an optical spectroscopy technique applied directly to the crystals prior to diffraction experiments. This would allow researchers to identify the best experimental conditions that induce specific spectroscopic signature changes and thus are most likely to induce specific structural changes.

### 9.2. Synchrotron or XFEL

Applications for XFEL beamtime are highly competitive owing to the small number of facilities that are present around the world. Beamtime for TR-SSX experiments will start to propagate in due course with the emergence of upgraded synchrotrons and beamlines allowing these types of experiments. One limitation of TR-SSX will be that it is reserved to probing timescales of microseconds and above, while TR-SFX will continue to allow timescales starting at hundreds of femtoseconds to be probed. However, most enzymes have a turnover in the millisecond-to-second range (Bar-Even *et al.*, 2011 ▸), making them adequate targets for TR-SSX experiments. This is especially true for mixing experiments, where small-molecule diffusion into crystals will always exceed microseconds and thus is well suited for TR-SSX. As a final point, specific radiation damage does not appear to be a major issue in TR-SSX experiments; however, experiments on particularly radiosensitive samples may still prove to be challenging, for which TR-SFX would continue to be the safer option.
